# Exposure of formal and informal nail technicians to organic solvents found in nail products

**DOI:** 10.3389/fpubh.2023.1147204

**Published:** 2023-05-04

**Authors:** Goitsemang Keretetse, Gill Nelson, Derk Brouwer

**Affiliations:** Occupational Health Division, School of Public Health, Faculty of Health Sciences, University of the Witwatersrand, Johannesburg, South Africa

**Keywords:** volatile organic compounds, passive sampling, task-based monitoring, VOC profile, bystander exposure, adjusted TVOC

## Abstract

Nail technicians are exposed to volatile organic compounds (VOCs) emitted from nail products used in their daily work, which may cause adverse health effects. This study aimed to assess VOC exposure of nail technicians in the South African formal and informal sectors and to provide a task-based exposure assessment of different nail applications. Personal passive sampling was conducted on 10 formal and 10 informal nail technicians located in the northern suburbs of Johannesburg and the Braamfontein area, over 3 days. Real-time measurements were taken to determine task-based peak exposures. The number of clients serviced, working hours, type of nail application, type of ventilation, room volume, and carbon dioxide (CO_2_) concentrations, were also recorded. There were differences in the nail products used, the types of nail applications performed, the number of clients serviced, and breathing zones VOC concentrations of the formal and informal nail technicians. Some formal nail salons were equipped with mechanical ventilation while the informal nail salons relied on natural ventilation. CO_2_ concentrations were higher in the informal than the formal nail salons and increased during the course of the working day. Formal nail technicians were exposed to higher total volatile organic compounds (TVOC) concentrations than informal nail technicians, which may be due to the different nail application procedures as well as ‘background’ emissions from their co-workers—the bystander effect. Acetone was the predominantly detected VOC: the formal nail technicians were exposed to significantly higher TWA (8 h) concentrations [geometric mean (GM) 43.8 ppm, geometric standard deviation (GSD) 2.49] than were the informal nail technicians (GM 9.87 ppm, GSD 5.13). Methyl methacrylate among the informal nail technicians was measured at 89.7% detection frequency, far higher than that among the formal nail technicians (3.4%). This may be attributed to the observed popularity of acrylic nail applications in this sector. Nail applications involving soak-off gave rise to high TVOC peaks at the start of the nail application process. This is the first study to compare organic solvent exposures among formal and informal nail technicians and determine task-based peak exposures. It also brings attention to the often-overlooked informal sector of this industry.

## 1. Introduction

Nail care service, as part of the beauty industry, comprises both the formal and informal sectors in South Africa. The formal sector comprises formalized and registered businesses, some of which are franchises with formal employment structures, regulated working hours, and monthly salaries. The informal sector comprises nail salons that are not registered or regulated by the state, are mostly self-owned or part of an existing business such as a hair salon, and are categorized by irregular working hours and unstable wages (1, 2). No longer a new industry, the nail care industry has grown exponentially, both locally and internationally, with the US projecting growth in the number of licensed manicurists and pedicurists by more than 10% from 2018 to 2028 (3–5).

Nail technicians spend their working days providing nail care services, which include manicures, pedicures, nail polish applications, ultra-violet (UV) gel applications, acrylic nail applications, and nail extensions in the form of artificial nails. These applications make use of a wide variety of products, comprising a cocktail of chemical substances. Products include nail polishes, nail polish removers, nail hardeners, artificial nails, nail tip adhesives, artificial nail removers, and disinfectants (6). Chemical substances in the nail products are typically aromatics hydrocarbons (toluene, ethyl benzene, xylene), aldehydes (formaldehyde), esters (ethyl acetate, butyl acetate, vinyl acetate, methyl methacrylate (MMA), ethyl methacrylate (EMA)), ketones (acetone and methyl ethyl ketone), alcohols (ethanol, isopropyl alcohol, and n-butyl alcohol), plasticisers (dibutyl phthalate, triphenyl phosphate), and terpentines (pinene, limonene, camphor, menthol) (7–9). Many of these chemicals are associated with health effects from exposure via inhalation and skin contact, including cancers, allergies, and respiratory, neurologic and reproductive disorders (4, 7, 10–13). Both nail technicians and their clients are exposed to these chemicals.

In 1974, the United States Food and Drug Administration (US-FDA) banned the use of MMA in cosmetic products due to its sensitizing effects, and reported incidents of fingernail damage and deformity, as well as contact dermatitis. It was subsequently replaced with EMA (14). Despite 32 states in the US also banning MMA (15), several exposure assessment studies in nail salons have reported its presence (12, 16–19). Some nail polishes contain harmful substances such as toluene, dibutyl phthalate (DBP) and formaldehyde, commonly known as the ‘toxic trio’ (20). There was a move in the US during the early 2000s to reduce the use of such harmful substances in nail products, with some nail polishes declared to be DBP-free or free from the ‘toxic trio’ (3, 21). These actions created awareness and helped regulate the chemical composition and labeling of nail products. The challenge is in enforcing compliance since cosmetic products and ingredients do not need US-FDA premarket approval, with the exception of color additives (22). The onus is on the manufacturers to ensure that these harmful substances are excluded from their products and to provide labels listing the contents on their products, as well as safety data sheets to consumers and nail salons. In South Africa, the formal sector can standardize the purchase of products, using procurement structures and procedures, which reduces the risk of sourcing products that contain banned substances or are not adequately labeled. The informal sector salons and technicians, however, procure products that are readily available and affordable, and which might contain banned and unsafe chemicals. This practice was reported in a study in Uganda where nail salons used cheap cosmetics to keep costs low (23).

Various exposure assessment methods have been used to measure VOC exposures in nail salons, including passive samplers, active samplers, direct reading instruments, and combinations thereof. For example, some researchers have conducted personal exposure measurements within the worker’s breathing zone, covering the full shift or up to 80% of the shift (4, 7, 18); some have measured ambient concentrations at strategic locations within the nail salon to assess within salon variability (3, 11); and others have used area measurements with the sampler positioned as close as possible to the worker’s breathing zone (12, 17, 19, 24).

No studies assessing nail technicians’ exposures to VOCs in nail products have been conducted in South Africa until now. The objectives of this study were to assess exposures of nail technicians, in the formal and informal sectors of the nail industry, to VOCs emitted from nail products, and to provide a task-based exposure assessment of different nail applications provided in the salons.

## 2. Materials and methods

### 2.1. Study population

This study was conducted in nail salons in the City of Johannesburg. Ten nail salons represented the informal sector in the Braamfontein area and six nail salons represented the formal sector in the northern suburbs. The formal nail salons were franchises of one of the largest local beauty companies, while the informal nail salons comprised unregistered nail salons, some of which operated inside hairdressing salons. Ten nail technicians from 10 informal nail salons, and 10 from six formal salons agreed to participate in the study.

### 2.2. Personal exposure measurements

The data collection procedure started with a brief walkthrough of each nail salon to identify the types of ventilation, the layout of the salon, the location of the nail service station(s), and other services offered such as massages, waxing, and hairdressing. Personal exposure measurements were collected by means of diffusion/passive sampling (Radiello® Passive sampler, Sigma Aldrich). Before sampling, the sorbent cartridge was removed from its glass storage tube and inserted into the diffusive body, taking care not to touch the cartridge. The passive sampler was attached to the lapel of the participant in order to obtain a sample within the breathing zone. Sampling devices were deployed to measure at least 80% of the work shift, over a period of three consecutive days, for each nail technician. A total of 60 personal exposure measurements in the formal (*n* = 30) and informal (*n* = 30) nail salons were collected. Field blanks were also collected to check for contamination during transport. A data capture sheet was used to record information such as the time that the passive sampler was opened and donned, the number and types of nail applications performed, the duration of the nail application procedures, personal protective equipment used, and other control measures that were in place. At the end of the sampling period, the passive sampler was detached from the participant’s lapel, placed in the glass tube, capped, labeled and stored at <5°C.

### 2.3. Task-based monitoring

Task-based exposure measurements were carried out using a MiniRAE 3,000 Photoionization detector (PID; RAE Systems, Sunnyvale, CA) to measure peak exposures during specific nail application activities. The task-based exposure measurements are key to determining peak exposures in order to target interventions. The PID was used for continuous detection of TVOCs, using a 10.6 eV lamp (range 0.1–15,000 ppm). Prior to each day of sampling, the PID was calibrated according to the manufacturer’s recommendations, which included zeroing the instrument with zero-grade air (fresh air) and span calibration with a single concentration of 100 parts per million (ppm) isobutylene gas. The direct reading instrument was programmed to data log concentrations every minute. Measurements were taken as close as possible to the nail technician’s breathing zone during the full duration of the nail application.

Salon dimensions were measured with a laser distance measurer (Laser Measurer Model GLM 80; Bosch). Multiple air quality parameters were measured at each salon at the start and the end of the work day**—**indoor and outdoor. These included CO_2_, used as a proxy for ventilation in the nail salons, temperature, and relative humidity (IAQ-Calc Indoor Air Quality Meter Model 7,545; TSI, Inc.). Measurements were data logged at 1-min intervals. Outdoor measurements were collected within 50 m of the nail salon and 150 m from the road or carpark.

### 2.4. Chemical analysis

The sorbent from each sample was extracted using 2 ml of carbon disulfide (CS_2_). The sample was stirred and allowed to stand for 30 min. Gas chromatography with a flame ionization detector was used to quantify the concentrations of VOCs. Chromatographic separation was performed using a DB-624 column (0.25 mm ID, 30 m length, 1.4 μm film thickness) for quantification and a DB-WAX column (0.25 mm ID, 30 m length, 1.5 μm film thickness) for verification, with a split injection of 3 μl and split flow of 20 ml/min. The carrier gas was nitrogen at a constant pressure of 5 psi. The injector temperature was 220°C, and the detector temperature was 200°C. The following temperature program was used: oven initial temperature of 35°C (hold for 10 min), ramp at 5°C/min up to final isotherm of 175°C (hold for 7 min). The total time for the analysis was 45 min. Peak areas were extracted by MSD ChemStation macro program, adjusted for internal standards, and transferred to an excel spreadsheet.

The average concentration over the exposure time interval was calculated from the mass of the analyte found on the cartridge and exposure time, without introducing any corrective factor. Average concentration, C (μg/m^3^) over the entire exposure time was calculated as:


(1) $$C\left[\mu g.m^{-3}\right]=\frac{m\left[\mu g\right]}{Q_{k}\left[ml.\min^{-1}\right]*t\left[\min\right]}\;1,000,000$$

Where, *Qk* is the sampling rate, *m* is the mass of analyte in μg, and *t* is the sampling duration in minutes.

Based on the literature review, and information obtained from the labels of the products used and the ingredients list (although not always listed), the following compounds were quantified: ethanol, acetone, methyl ethyl ketone (MEK), ethyl acetate, benzene, trichloroethylene, methyl methacrylate, ethyl methacrylate, propyl acetate, methyl isobutyl ketone (MIBK), toluene, perchloroethylene, n-butyl acetate, ethyl benzene, xylene, d-limonene, chloroform, 2-propanol, 1-propanol, and white spirits. To simplify the calculation, the limit of detection for all compounds was set at 0.50 μg/ml and, the average lowest concentration value of the samples was set at 0.004 ppm.

### 2.5. Data analysis

Data pre-processing and visualization were conducted using Microsoft Excel 2016. Data were analyzed using the following statistical tool packages and calculators: ExpoStats, a Bayesian Toolkit (25), BWStat (version 2.1; Belgian Society for Occupational Hygiene), IHMod (version 2.0, AIHA, 2021), and Stata software (version 17; StataCorp LLC, Texas, USA). Normality of VOC concentrations was assessed using the Expostats NDistrib tool for assessing goodness of fit for the lognormal and normal distributions. Thirteen VOCs with a detection frequency of ≥30% were selected from the 20 reported VOCs for further analysis. Descriptive statistics, including geometric means and standard deviations, and interquartile ranges, were calculated using ExpoStats. Given that the type and number of VOCs differed within and between the two sectors, with some of the VOCs having much higher concentration compared to others. Summing the concentrations would result in skewed results. Therefore the adjusted total VOC concentration was calculated to enable comparison. Adjusted TVOC concentration was calculated using the 13 detected VOCs, after correcting by their respective evaporation rate relative to the evaporation rate of d-limonene (the VOC with the lowest evaporation rate; Supplementary Table S1). The evaporation rates were calculated by means of Hummel’s equation (26), using the IHMod 2.0 mathematical model supporting files. The ratio of the evaporation rate of each VOC and that of d-limonene was used to correct the concentration of each individual VOC in what is referred to as the weighted concentration. The sum of these individual VOC weighted concentrations was the adjusted TVOC concentration in mg/m^3^. The f-test was used to calculate variance and the independent t-test to calculate the difference in adjusted TVOC between the formal and informal nail technicians.

Imputation for data below the limit of detection (LoD) was performed for the VOC concentrations, using Expostats-NDexpo/RoS tool (9, 27). NDexpo is a web application that implements a rigorous censored data treatment method, the regression on order statistic (Robust ROS), as described by Helsel (28). This approach is based on fitting detected values to a lognormal distribution and then predicting a replacement value for each nondetect as a function of the fitted distribution, and its rank among the detected values. The analytical LoD and LoQ of 0.50 and 1.65 (3.3 times LoD) microgram/ml, respectively, were converted into a LoQ sampling concentration of 0.0132 ppm. The independent t-test was used to investigate differences between formal and informal nail technicians, using the natural log (ln) transformed concentrations of the six VOCs with ≥80% of all observation above LoD.

Associations between VOC concentrations and other variables were evaluated using Pearson’s correlation coefficient. The variability of acetone concentrations, specifically within- and between-salon variability, and within- and between nail technicians in the respective sectors, were evaluated using a one-way analysis of variance (ANOVA) test, implemented in BWStat version 2.1, after ln-transforming the data. Task-based measurements were graphically represented as time series plots, showing detailed phases of each reported nail application. These were only used as qualitative indications of TVOC exposure peaks during a specific nail application.

The differences in the indoor CO_2_ concentrations over the workday (ΔCO_2_) were determined by calculating the difference between outdoor and indoor CO_2_ concentration at the start and the end of the day. This is a similar approach used by Alaves (17) who calculated the change in CO_2_ (“CO_2_ differential”), i.e., the difference in pre- and post-sampling CO_2_ measurements. The ΔCO_2_ was correlated against the number of clients serviced.

The study was approved by the University of the Witwatersrand Human Research Ethics Committee (HREC): certificate number M171184.

## 3. Results

### 3.1. Nail salon characteristics

All nail salons in this study offered basic nail services, including manicures and pedicures, nail polish applications, gel applications, and acrylic applications. Most of the nail applications began with an acetone soak-off process to remove existing nail applications. Different methods of nail polish removal, referred to as soak-off, were observed. The formal nail technicians most commonly administered the soak-off by immersing the client’s nails in an acetone hot bath. This is done by filling a bowl (large enough for the client’s fingers to fit) with acetone and placing it inside a larger bowl filled with hot water. This heats the acetone slightly, which speeds up the nail application removal process. The method is quick and reduces the duration of the nail application process. The informal nail technicians made use of cotton wool pads soaked in acetone, which were applied to the client’s nails, and wrapped in tin foil. This method takes longer but uses less acetone than the hot bath method.

The most common applications in the formal and informal nail salons were gel and acrylic nail applications, respectively. Formal nail salons used similar products from known brands, procured through a structured company system, while informal nail salons used a variety of brands and suppliers.

Table 1 shows a comparison of basic characteristics of environments in the formal and informal salons. Formal nail technicians provided nail services to more clients (5.8 ± 1.7) than did informal nail technicians (3.3 ± 2.0). However, the mean number of working hours per day was higher in the informal nail salons (9.7 ± 1.2 hours). Together, this suggests that the mean duration per application was longer in the informal than the formal nail salons. The mean room volume of the formal nail salons (154 m^3^) was almost twice that of the informal nail salons (86.1 m^3^). All the informal nail salons were located in hair salons opening to the roadside, while five (83.3%) of the formal nail salons were located in a shopping center/strip mall with the main door opening to a carpark; only one formal nail salon was located inside a shopping mall with a central walkway where retail stores face one another. Informal nail salons relied predominantly on natural ventilation through open doors (no windows), while nail salons in the formal sector made use of both natural and mechanical ventilation in the form of heating, ventilation and cooling (HVAC) systems. While all participating formal nail salons had an HVAC system, only two (33.3%) used it during the sampling period. In general, salons that were not using their HVAC systems relied on natural ventilation by opening their doors. None of the participating salons had local extraction ventilation (LEV) systems. Mean temperature and relative humidity in the informal nail salons were higher (AM 26°C, SD 1.4 and AM 53%, SD 7.5, respectively) than those in the formal nail salons (AM 24°C, SD 2.4 and AM 39%, SD 13.6, respectively). Informal nail salons had, on average, higher indoor CO_2_ concentrations (AM 713 ppm, SD 299) than the formal nail salons (AM 492 ppm, SD 119) at the end of the working day. The mean ΔCO_2_ in the formal nail salons showed an increase in CO_2_ during the course of the day (AM 35 ppm, SD 176) while, in the informal nail salons, there was a decline (AM −52 ppm, SD 400).

**Table 1:** Characteristics of formal and informal nail salon environments.

Variable	Formal nail salons (*n* = 6)					Informal nail salons (*n* = 10)				
	*n*	%	AM	SD	IQR	*n*	%	AM	SD	IQR
Number of clients serviced			5.8	1.7	4–7			3.3	2.0	2–4
Working hours per day (hours)			8.6	0.5	8–9			9.7	1.2	9–10
Salon location										
Roadside	0	0				10	100.0			
Shopping center/strip mall	5	83.3				0	0			
Shopping mall	1	16.7				0	0			
Ventilation										
Natural ventilation	4	66.6				10	100.0			
Mechanical general ventilation (HVAC)	6	100.0				0	0			
HVAC system operational	2	33.3				0	0			
Room volume (m^3^)			154	12.8	151–162			86.1	37.8	56.4–95.0
CO_2_ (ppm)*			492	119	404–551			713	299	522–887
ΔCO_2_ (ppm)			35	176				−52	400	
Temperature (°C)			24	2.4	23–26			26	1.4	26–28
Relative humidity (%)			39	13.6	25–49			53	7.5	48–57

^*^Average indoor CO_2_ levels at the end of the day.

AM, arithmetic mean; SD, standard deviation; IQR, interquartile range; HVAC, heating, ventilation and cooling system.

### 3.2. Concentration levels of organic solvents

Sixty personal exposure measurements were collected in the formal (*n* = 30) and informal (*n* = 30) nail salons. One informal study participant did not present for work on the third day of the assessment, and all measured VOCs for one formal participant were below the limit of detection for day 3 of the assessment. The latter scenario may have been a result of mishandling the sampling device during the measurement period. Thus, two samples were excluded from the analysis, resulting in a sample size of 58, i.e., 29 in each group. Field blanks showed negligible VOC levels, confirming that transport, storage, and handling activities did not contaminate the sampling device.

**Figure 1:**
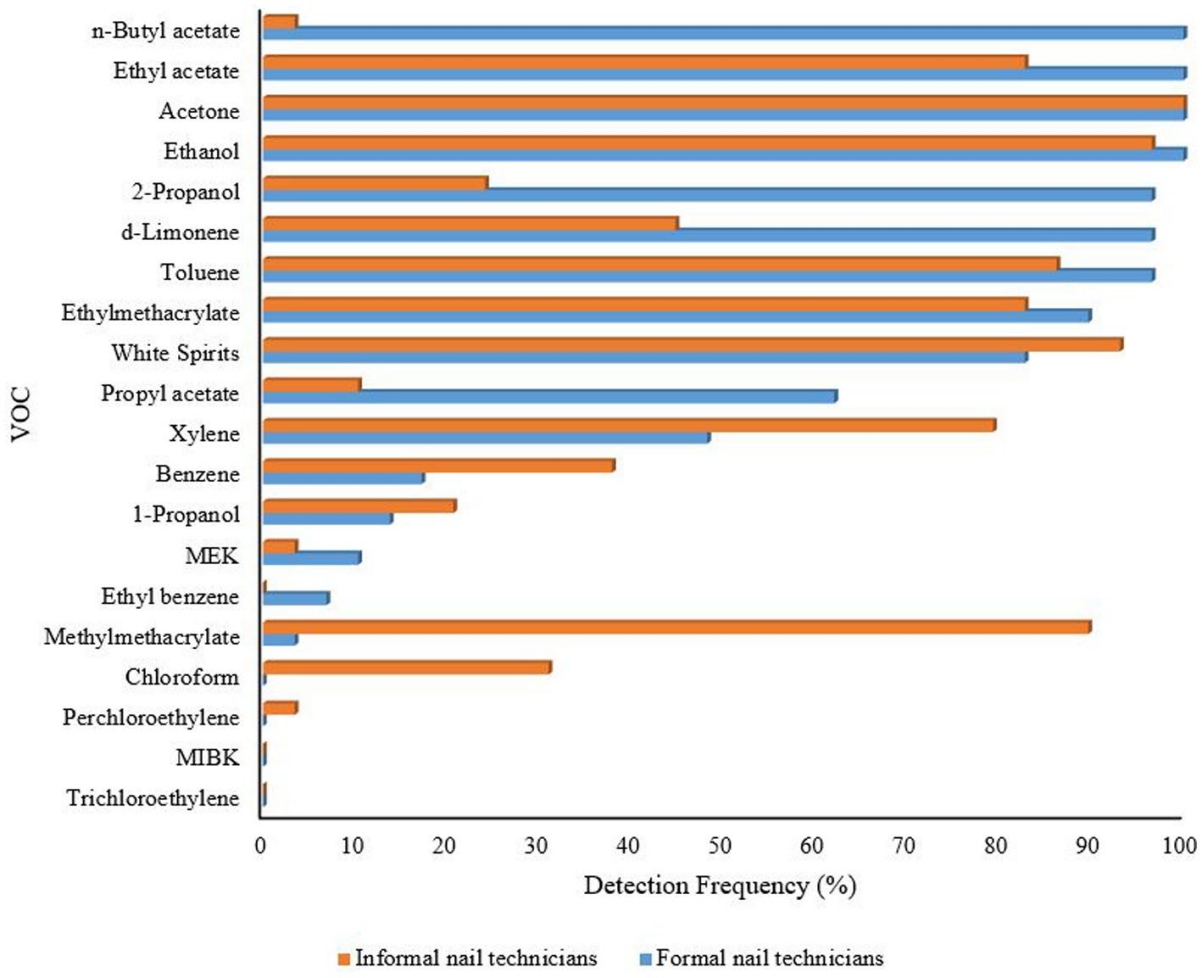
Profile of all volatile organic compounds (VOCs) detected in shift-based measurements among formal and informal nail technicians.

The chemical profiles of the full shift personal exposure sampling of the formal and informal nail technicians are shown in Figure 1. Of the 20 VOCs reported, 13 showed a detection frequency of ≥30% the LoD, *viz.* n-butyl acetate, ethyl acetate, acetone, ethanol, 2-propanol, d-limonene, toluene, ethyl methacrylate, methyl methacrylate, white spirits, propyl acetate, xylene, and benzene.

As shown in Table 2, VOC concentrations differed between the formal and informal nail salons. Six of the detected VOCs, namely ethanol, acetone, ethyl acetate, ethyl methacrylate, toluene and white spirits, had detection frequency (DF) of 80% above the LoD in both the formal and informal nail salons. For formal nail technicians, the GM of the concentrations for acetone, ethyl acetate and ethanol that presented with 100% DF was significantly higher than that for the informal nail technicians, where the GSDs were much higher. A significant difference was also seen in white spirit concentration between the two groups (*p* < 0.05). Noticeably, a 100% DF was observed for n-butyl acetate among the formal nail technicians, whereas this substance was detected in only one of the 29 samples (3.4%) among the informal nail technicians. Methyl methacrylate was found in 89.7% of the samples among the informal nail technicians compared to only one sample (3.4%) among the formal nail technicians.

**Table 2:** Summary of volatile organic compounds (VOC) concentrations (ppm) in the formal and informal nail salons.

VOC	Formal nail salons (*n* = 6)					Informal nail salons (*n* = 10)					*p*-value*
	*n*	%	GM	GSD	IQR	*n*	%	GM	GSD	IQR	
Ethanol	29	100.0	2.06	1.58	1.68–2.66	29	96.6	1.24	4.4	0.633–2.92	<0.05
Acetone	29	100.0	43.80	2.49	26.3–66.1	29	100.0	9.87	5.13	4.59–32	<0.05
Ethyl acetate	29	100.0	0.559	1.72	0.406–0.868	29	82.8	0.058	5.44	0.023–0.16	<0.05
Benzene	29	17.2	0.004	1.33	0.004–0.005	29	37.9	0.003	2.33	0.002–0.005	
Methyl methacrylate	29	3.4	0.002	–	–	29	89.7	1.970	9.48	0.588–13.3	
Ethyl methacrylate	29	89.7	0.334	6.93	0.083–1.21	29	82.8	0.765	14.8	0.0748–7.03	>0.05
Propyl acetate	29	62.1	0.007	3.12	0.003–0.016	29	10.3	0.004	2.23	0.002–0.006	
Toluene	29	96.6	0.010	1.81	0.007–0.015	29	86.2	0.008	1.56	0.006–0.010	>0.05
n-Butyl acetate	29	100.0	0.131	1.97	0.085–0.202	29	3.4	0.002	–	–	
Xylene	29	48.3	0.005	2.64	0.003–0.009	29	79.3	0.006	1.69	0.005–0.008	
d-Limonene	29	96.6	0.025	2.75	0.015–0.038	29	44.8	0.004	6.99	0.001–0.003	
2-Propanol	29	96.6	17.80	1.72	13.7–23.6	29	24.1	0.139	6.16	0.041–0.416	
White Spirits	29	82.8	0.076	4.81	0.017–0.204	29	93.1	0.155	2.45	0.084–0.288	<0.05

*n*, sample number; %, detection frequency; GM, geometric mean; GSD, geometric standard deviation; IQR, interquartile range. *Student *t*-test.

**Figure 2:**
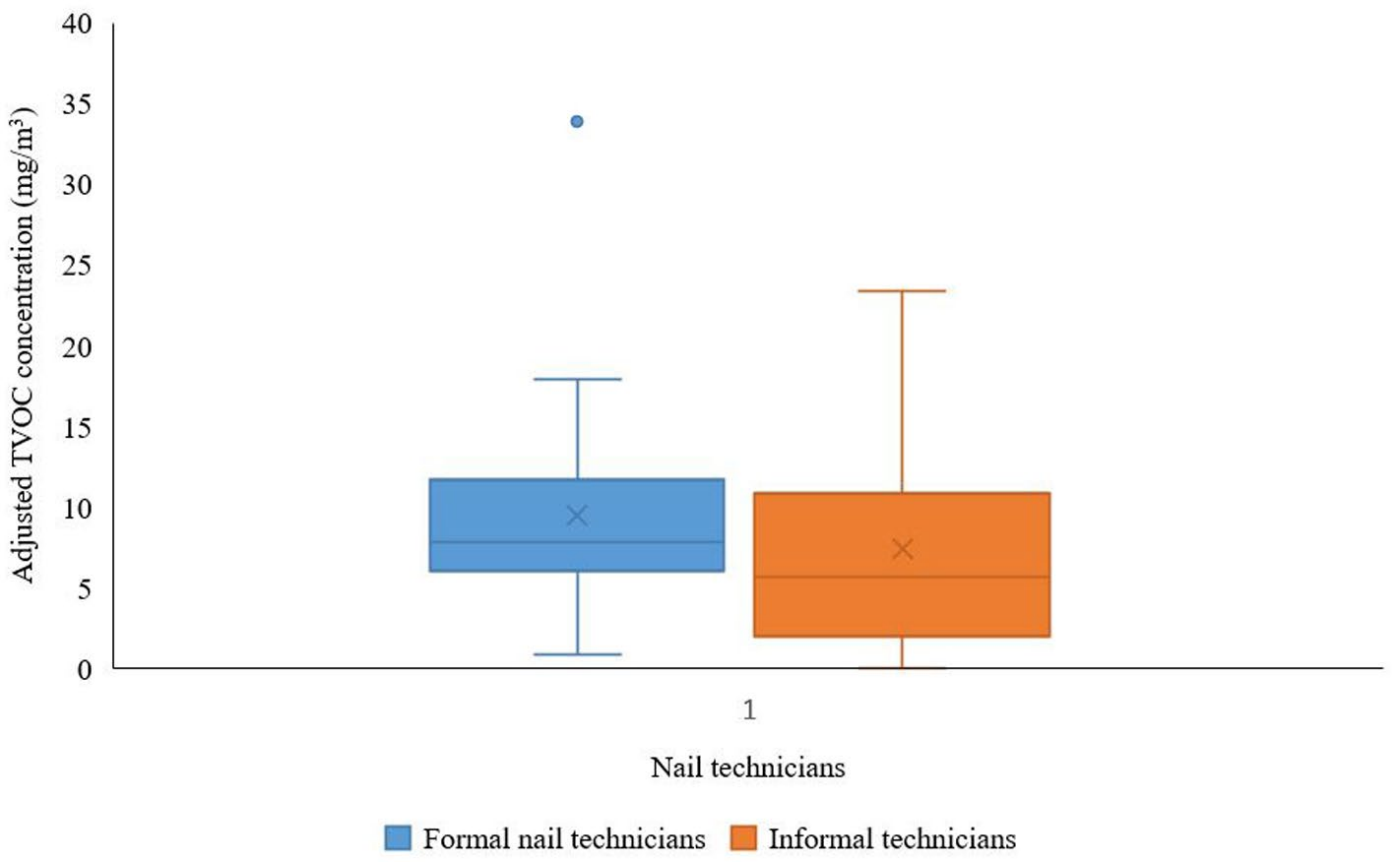
Comparison of the adjusted total volatile organic compounds (TVOC) concentrations of 13 measured solvents detected in formal and informal nail technicians. In the box plots, the boundary of the box closest to zero indicates the 25th percentile (1st quartile), the line within the box marks the median, the “*x*” within the box marks the mean, and the boundary of the box farthest from zero indicates the 75th percentile (3rd quartile). Whiskers above and below the box indicate the upper and lower extremes. The circle above the whiskers indicates the outlier or the value greater than 1.5 the value of the third quartile.

Figure 2 shows box and whisker plots of the distribution of VOC concentrations in the formal and informal nail salons, corrected for their respective evaporation rates (Supplementary Table S1). The formal nail technicians had a higher adjusted mean TVOC (GM 7.24 mg/m^3^, GSD 1.91) than the informal nail technicians (GM 4.12 mg/m^3^, GSD 3.84). There was no statistically significant difference in the adjusted TVOCs between formal and informal nail technicians. Although acetone had the highest concentrations in both groups, it contributed only 39.9 and 13.6% to the adjusted TVOC concentrations in the formal and informal sectors, respectively. With 46.9% contribution to the TVOCs, 2-propanol was the dominant VOC in the formal nail salons, whereas both MMA an EMA contributed the most to the TVOCs in the informal sector, *viz.* 39.3 and 33.3%, respectively.

One-way ANOVA-tests were conducted to compare the variability of acetone**—**the VOC with the highest concentrations**—**between and within nail salons in the formal and informal sector (Supplementary Tables S2, S3). The acetone concentration differed significantly, between the informal nail salons [*F*(9, 19) = 8.88, *p* < 0.001], and between the formal nail salons [*F*(5, 23) = 4.08, *p* = 0.008].

Table 3 shows the Pearson’s correlation coefficients for selected VOCs and some of the salon characteristics, i.e., number of clients serviced, and ΔCO_2_. The VOC contributing the most to the adjusted TVOC in the formal sector—2-propanol—was included in the correlation analysis. Concentrations of ethanol, acetone, and ethyl methacrylate showed a significant correlation with the number of clients serviced among the informal nail technicians (*p* < 0.05, Table 3).

**Table 3:** Pearson’s correlation coefficients and value of ps between volatile organic compounds (VOCs) concentration, number of clients seen per day and ΔCO_2_ among formal and informal nail salons.

VOC	Formal				Informal			
	No. of clients		ΔCO_2_		No. of clients		ΔCO_2_	
	*r*	*p*-value	*r*	*p*-value	*r*	*p*-value	*r*	*p*-value
Ethanol	0.023	0.902	−0.124	0.521	0.375*	0.045	0.219	0.543
Acetone	−0.181	0.347	0.131	0.499	0.562*	0.002	−0.171	0.636
EA	−0.021	0.912	−0.364	0.052	0.017	0.931	0.076	0.835
EMA	−0.087	0.652	−0.246	0.198	0.663*	0.000	−0.092	0.801
Toluene	−0.234	0.222	0.019	0.921	−0.065	0.738	0.247	0.491
2-Propanol	0.173	0.370	−0.309	0.103	0.239	0.213	0.104	0.776
White spirits	−0.157	0.418	−0.049	0.802	0.259	0.176	−0.009	0.979

EA, ethyl acetate; EMA, ethyl methacrylate. ΔCO_2_, the difference in the indoor CO_2_ concentrations over the workday.

As shown in Supplementary Table S4, there were strong and significant correlations between ethyl methacrylate and ethanol (*r* = 0.77; *p* ≤ 0.01), 2-propanol and ethanol (*r* = 0.80; *p* ≤ 0.01), and 2-propanol and ethyl methacrylate (*r* = 0.55; *p* ≤ 0.01). Acetone, ethyl methacrylate, white spirits, and the number of clients serviced were negatively correlated with ΔCO_2_, which ranged from −0.01 to −0.17, for the formal nail salons (Table 3). In the formal nail salons (Supplementary Table S5), there were strong and significant correlations between ethyl acetate and ethanol (*r* = 0.55; *p* = <0.01), toluene and acetone (0.87; *p* = <0.01), 2-propanol and ethanol (*r* = 0.53; *p* ≤ 0.01), and 2-propanol and ethyl acetate (*r* = 0.58; *p* ≤ 0.01). Negative correlations were observed in the formal nail salons between the concentrations of ethanol, ethyl acetate, ethyl methacrylate, 2-propanol, white spirits, and ΔCO_2_.

### 3.3. Task-based exposures

**Figure 3:**
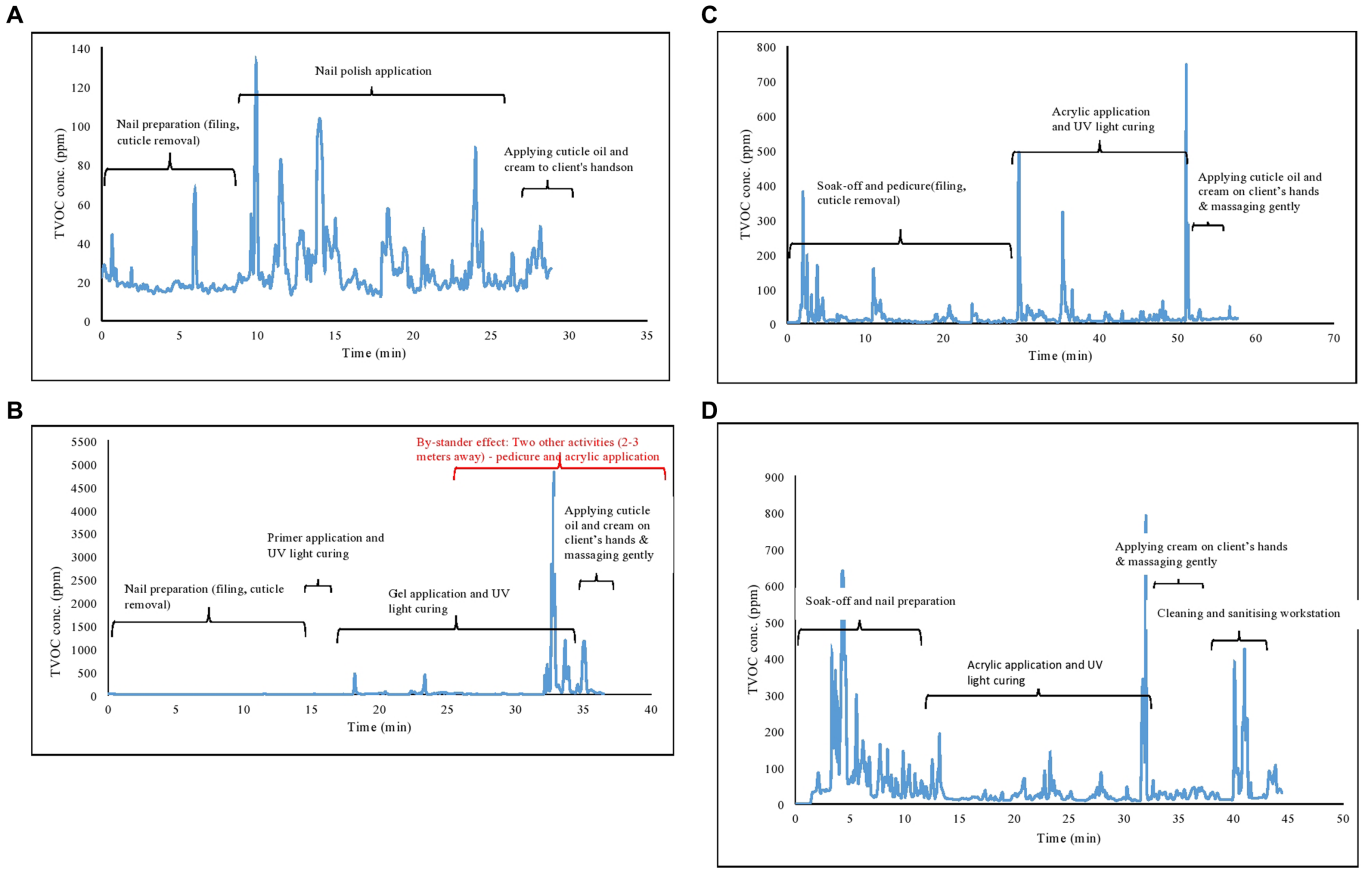
**(A–D)** Time profiles of peak exposure to volatile organic solvents (ppm) of various nail applications in the formal nail salons **(A)**: buff and paint; **(B)**: gel application; **(C)**: soak off, pedicure and gel application; **(D)**: soak off and acrylic application.

**Figure 4:**
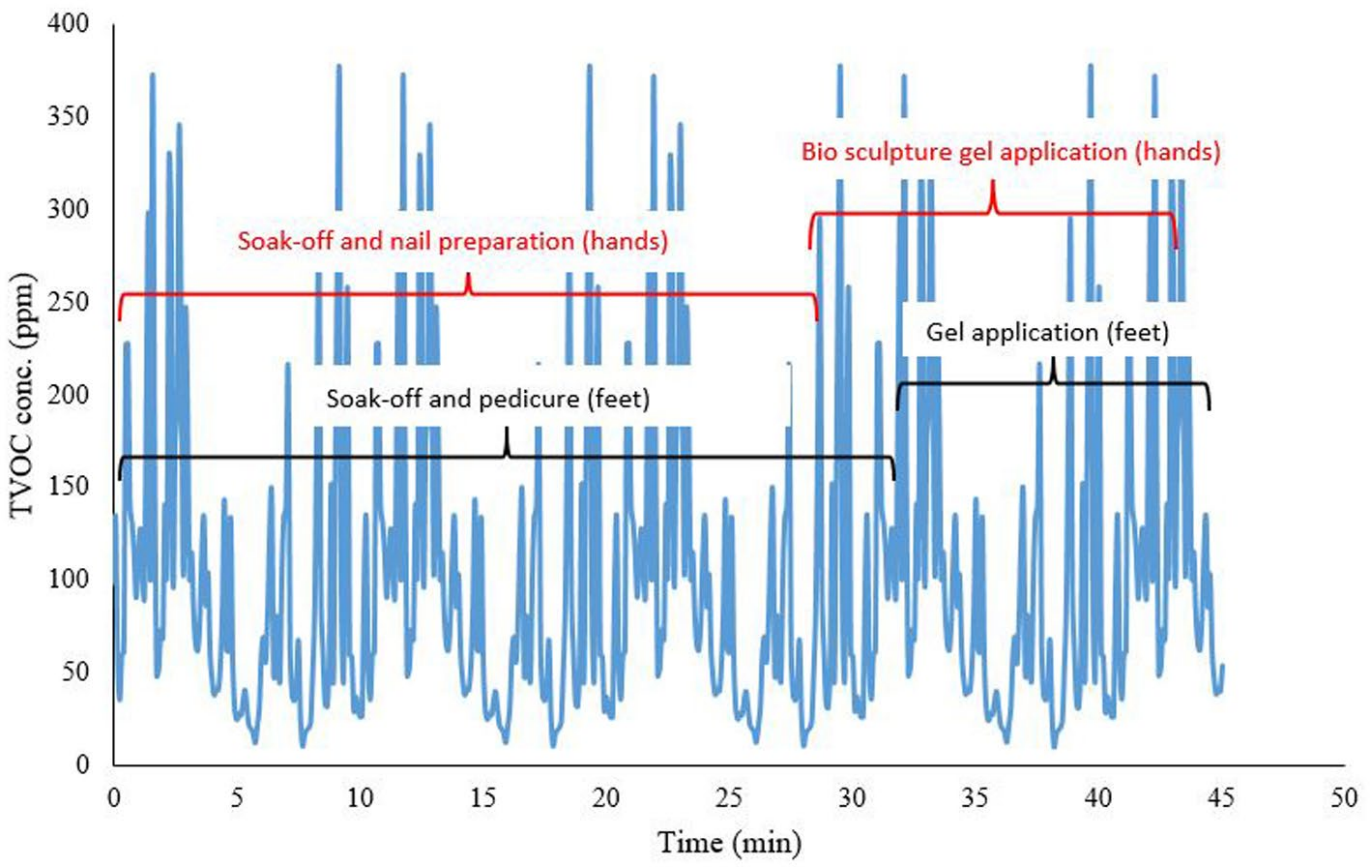
Time profile of peak exposure to volatile organic solvents (ppm) of soak off and bio sculpture gel application on hands and pedicure, simultaneously, in the formal nail salons.

Figure 3 shows time profiles of peak exposures to volatile organic solvents (ppm) of various nail applications (a: nail polish application (buff and paint), b: gel application; c: soak off pedicure and gel application; d: soak off and acrylic application) in the formal nail salons. Typical nail applications performed in a formal nail salon are basic manicures and pedicures, UV gel or gelish applications, bio-sculpture applications, normal paint applications (also referred to as buff and paint), and acrylic applications. Sometimes, two nail technicians work simultaneously on one client, conducting multiple nail applications (Figure 3B). Figure 4 shows soak-off and bio sculpture applications on a client’s hands, and pedicure, provided simultaneously by two nail technicians.

## 4. Discussion

The objective of this study was to assess the breathing zone concentration of key organic solvents generated from nail products during nail application services, among formal and informal nail technicians. We also assessed task-specific exposures to identify short duration and peak exposure patterns.

Differences were noted regarding the basic characteristics of the nail salons. All the formal nail salons were fitted with mechanical ventilation in the form of ceiling exhaust fans and HVAC systems, although only two nail salons (33.3%) operated their ventilation systems at the time of sampling. All informal nail technicians, on the other hand, relied on natural ventilation (open doors). The rooms of the formal nail salons were larger, by volume, than those of the informal nail salons. Interesting to note is that the adjusted TVOC concentration was higher in the formal than the informal nail salons where only natural ventilation was used. Goldin et al. (29) reported that TVOC concentrations were inversely associated with ventilation, where salons with poorer ventilation had higher CO_2_ concentrations and significantly higher TVOC concentrations. Park et al. (7), however, reported that concentrations measured in salons with general mechanical ventilation systems were higher than those without the systems. They speculated that the ventilation system, especially the ceiling exhaust duct, may have contributed to the spread of chemicals in the room and toward the worker’s breathing zone. Persily (30) proposed using CO_2_ as an indicator or metric of outdoor ventilation rates (30). This metric takes into consideration that indoor CO_2_ depends on several factors, including the occupants’ rate of generating CO_2_, outdoor air ventilation rates, time of occupancy, and outdoor CO_2_ concentrations.

In our study, we calculated the ΔCO_2_ and found that the CO_2_ concentrations decreased during the day in the formal nail salons but increased in the informal nail salons, suggesting that the formal salons had better ventilation. The informal nail salons had a higher mean CO_2_ concentration than the formal nail salons. Important also to note is that the ΔCO_2_ is also affected by the number of occupants (clients and technicians) in the room. The number of clients serviced differed between the formal and informal nail salons. From our observations, the formal nail technicians serviced, on average, more clients—almost twice the number serviced by the informal nail technicians—and, therefore carried out more nail applications.

For the purpose of comparison between the formal and informal sectors, and to adjust for overweighting the results of the relatively higher volatile VOCs, we developed the concept of adjusted TVOC where the selected VOC concentrations were adjusted for their specific evaporation rate. Adding all adjusted VOC concentrations resulted in an adjusted TVOC concentration as a metric for cumulative exposure to the VOCs detected and included in our analysis. Our approach, i.e., a surrogate d-limonene equivalent concentration, is quite comparable with the toluene-equivalent concentration as deployed by Wallenius et al. (31). Differences were noted in adjusted TVOC concentrations between the formal and informal nail salons, although not significant. The formal nail salons had a higher adjusted TVOC concentration than the informal nail salons. During the COVID-19 pandemic and the national lockdown restrictions, the informal nail salons experienced a reduction in the number of clients visiting their salons, resulting in fewer of clients serviced per day than the period before the pandemic, which could explain the large variation in VOC concentrations. The formal sector also had several nail technicians working in each salon (four to six), while most of the informal nail technicians worked alone or in pairs in a hair salon. Nail services performed by co-workers and associated VOC emissions in the formal nail salons may have contributed to the relatively high TVOCs (i.e., bystander exposure) with relatively low variation.

Of the 20 VOCs measured, 13 showed a DF of ≥30% above the limit of detection (LoD), similar to findings from other studies (3, 7, 12, 17–19). However, the measured concentrations were all below the respective South African occupational exposure limits (OEL), and the ACGIH threshold limit value (TLV). Acetone was detected at 100% DF in both the formal and informal nail salons with concentrations significantly higher among the formal nail technicians. Similarly high levels of acetone were measured in studies by Park et al. (7) and Ma et al. (4). Acetone is the main ingredient in nail polish removers and is used in all nail treatments to remove existing nail applications. The preferred method of soak-off may have contributed to the high concentrations of acetone in the formal sector and to the difference between the adjusted TVOC in the formal and informal sectors, since acetone was one of the VOC contributing the most to the shift-based TVOC concentrations. Between nail technician variations from the one-way ANOVA showed significant differences in acetone concentrations between nail technicians in the formal and those in the informal nail salons. Factors mentioned above, i.e., number of clients serviced, type of nail applications conducted, duration of the nail application, as well these the acetone soak-off method performed, played a role in these observed variations.

Methyl methacrylate was detected among the informal nail technicians and, to a far lesser extent, among the formal nail technicians. This may be attributed to the greater popularity of the acrylic nail application method among clients in the informal sector. Another contributing factor may be the use of products in the informal sector which still contain MMA. Small quantities of MMA have also been detected in some of the airborne VOC measurements in other studies (7, 17, 18, 32). Although the US-FDA has banned the use of MMA (22), in some countries, including South Africa, products containing MMA are still used (12, 16, 17).

Toluene, one of the ‘toxic trio’ (20) was detected in most of the formal and informal nail salons, but at low concentrations. Similar low concentrations have been detected in other studies (3, 4, 17–19). These low levels may be indicative of efforts to move towards ‘toxic trio’ free nail products (3).

n-Butyl acetate, an ingredient in nail polish and some nail polish removers, was detected at 100% DF for the formal nail technicians but at only 3.4% DF (1 observation) for the informal nail technicians. 2-propanol, was detected at 96.6% DF among the formal nail technicians and was the highest contributor, followed by acetone, to the adjusted TVOC for the formal nail technicians. This may be related to the frequency of nail applications conducted in the formal sector, using products that contain n-butyl acetate, as well as the practice of applying disinfectant (containing 2-propanol) during the nail preparation process (as observed in the formal sector). The method of applying nail polish remover during the soak-off, may also be a contributing factor.

The correlations between the VOCs themselves in the formal and informal sector is indicative of a relationship between the emissions and the type of application performed. The formal sector VOCs are most likely generated from the nail polish application and soak-off method, while informal sector VOC emissions are related to acrylic applications. The correlations between the number of clients and concentrations of three VOCs (ethanol, acetone, and ethyl methacrylate) were stronger in the informal than the formal sector. It can be assumed that the concentrations of the VOCs in the breathing zone of the informal technicians can be attributed primarily to emissions during the treatments. In the formal sector, VOC concentrations may be increased by emissions from other nail technicians simultaneously conducting treatments in the same space.

Task-based measurements (real-time TVOC measurements) for short-term peak exposures were conducted to provide additional exposure characteristics beyond the shift-based personal exposure measurements. High TVOC peaks were measured during nail applications that included soak-off at the beginning of the nail application process to remove existing nail polish. Acrylic applications emitted higher levels of TVOC concentrations than other nail applications. Some of the nail applications were conducted simultaneously (hands and feet). This saved time since two nail applications could be conducted at the same time, but exposure was potentially increased. There was also the “bystander exposure” effect where simultaneous applications were conducted. Since there was a co-worker conducting a pedicure and another conducting an acrylic application within 2 m, it is likely that the combined emissions contributed to the measured concentrations.

There were several limitations to our study. First, we used convenience sampling to select study participants. Consequently, we cannot generalize the results. However, we have shown that exposures to VOCs in the nail industry might be high enough to potentially cause adverse health effects. Second, the salon room occupancy was not monitored throughout the entire measurement period and thus occupants’ contribution to the CO_2_ concentrations could not be determined. Since CO_2_ concentrations are affected by both occupancy and ventilation, the differences in CO_2_ concentrations alone have limited value as a proxy of room ventilation in the nail salons. Nevertheless, from observations, we believe that the increase in CO_2_ during the day that was seen in the informal nail salons is indicative of a lack of proper ventilation. A third limitation is the sensitivity of the PID in characterizing individual VOCs. Only the individual 20 VOCs for the 8-h personal exposure measurements were identified, but these could not be directly used to determine the chemical composition of the vapors detected during the task-specific measurements. Lastly this study did not involve biological monitoring to determine the internal dose.

Regardless of these limitations, this is the first study to assess exposure to VOCs in the often-overlooked informal sector, and to compare exposures with the formal sector of the beauty industry. The combination of shift measurements and task-based measurements is particularly useful for providing an exposure profile in this under researched group of workers and identifying high-exposure tasks for targeted interventions.

## 5. Conclusion

The study findings highlight the potential exposures of informal sector workers in the beauty industry, which are often overlooked. We found differences in the VOCs emitted from the nail products used, the nail applications performed, the number of clients serviced, and the VOC concentrations measured within the breathing zones of both the formal and informal nail technicians in this study. The formal nail salons had ventilation systems and more spacious rooms than the informal nail salons. More nail technicians worked in the formal nail salons at any one time, and more clients were serviced per salon, in comparison to the informal nail technicians. These factors played a role in the measured VOC concentrations and exposures: the formal nail technicians were exposed to higher TVOC concentrations, which may be due to the specific ‘soak off’ procedures used as well as ‘background’ emissions from their co-workers. The differences in the profiles of identified VOCs between the two sectors indicates the importance of the selection and procurement of ‘safe’ nail treatment products.

Studies of this nature are useful in developing intervention strategies to minimize exposure, such as informed decision making with regard to procurement of nail products, and adoption of safe work practices to reduce emissions from harmful chemicals among nail salon workers and their clients. The findings also inform policy development to help regulate these industries and ensure worker health and safety.

## Data availability statement

The raw data supporting the conclusions of this article will be made available by the authors, without undue reservation.

## Ethics statement

The studies involving human participants were reviewed and approved by University of the Witwatersrand Human Research Ethics Committee (HREC). The patients/participants provided their written informed consent to participate in this study.

## Author contributions

GK, DB, and GN: conceptualization and design of the study. GK: literature review, original draft of manuscript, and data collection. GK, DB: data analysis and interpretation. DB and GN: critical review and editing of the manuscripts. All authors contributed to the article and approved the submitted version.

## Funding

This work was supported by the South African National Research Foundation (NRF) grant number TTK170511230614, and the South African Department of Higher Education and Training New Generation of Academics Program (nGAP) fellowship fund.

## Conflict of interest

The authors declare that the research was conducted in the absence of any commercial or financial relationships that could be construed as a potential conflict of interest.

## Publisher’s note

All claims expressed in this article are solely those of the authors and do not necessarily represent those of their affiliated organizations, or those of the publisher, the editors and the reviewers. Any product that may be evaluated in this article, or claim that may be made by its manufacturer, is not guaranteed or endorsed by the publisher.

## References

[1] Chen MA. The Informal Economy—Theories, Definitions and Policies. Manchester, UK: WIEGO working paper no 1 (2012).

[2] Statistics South Africa. (2021). Quarterly labour force survey 1:2021. Pretoria, South Africa: Statistics South Africa. Available from: http://www.statssa.gov.za/publications/P0211/P02111stQuarter2021.pdf (Accessed 10, Jun 2021).

[3] Harrichandra A Roelofs C Pavilonis B. Occupational exposure and ventilation assessment in New York City nail salons. Ann Work Exposures Health. (2020) 64:468–78. doi: 10.1093/annweh/wxaa035, PMID: 32266385

[4] Ma GX Wei Z Husni R do P Zhou K Rhee J. Characterizing occupational health risks and chemical exposures among Asian nail salon workers on the East Coast of the United States. J Community Health. (2019) 44:1168–79. doi: 10.1007/s10900-019-00702-0, PMID: 31297649 PMC6913878

[5] Nguyen LV Diamond ML Kalenge S Kirkham TL Holness DL Arrandale VH. Occupational exposure of Canadian nail salon workers to plasticizers including phthalates and organophosphate esters. Environ Sci Technol. (2022) 56:3193–203. doi: 10.1021/acs.est.1c04974, PMID: 35156803

[6] Madnani NA Khan KJ. Nail cosmetics. Indian J Dermatol Venereol Leprol. (2012) 78:309–17. doi: 10.4103/0378-6323.95445 22565430

[7] Park SA Gwak S Choi S. Assessment of occupational symptoms and chemical exposures for nail salon technicians in Daegu City, Korea. J Prev Med Public Health. (2014) 47:169–76. doi: 10.3961/jpmph.2014.47.3.169, PMID: 24921020 PMC4050214

[8] Ceballos DM Craig J Fu X Jia C Chambers D Chu MT. Biological and environmental exposure monitoring of volatile organic compounds among nail technicians in the greater Boston area. Indoor Air. (2019) 29:539–50. doi: 10.1111/ina.12564, PMID: 31112343 PMC6565444

[9] Craig JA Ceballos DM Fruh V Petropoulos ZE Allen JG Calafat AM. Exposure of nail salon workers to phthalates, Di(2-ethylhexyl) terephthalate, and organophosphate esters: a pilot study. Environ Sci Technol. (2019) 53:14630–7. doi: 10.1021/acs.est.9b02474, PMID: 31736299 PMC7192361

[10] White H Khan K Lau C Leung H Montgomery D Rohlman DS. Identifying health and safety concerns in southeast Asian immigrant nail salon workers. Arch Environ Occup Health. (2015) 70:196–203. doi: 10.1080/19338244.2013.853644, PMID: 25965322

[11] Pavilonis B Roelofs C Blair C. Assessing indoor air quality in new York City nail salons. J Occup Environ Hyg. (2018) 15:422–9. doi: 10.1080/15459624.2018.1447117, PMID: 29494285 PMC8974398

[12] Zhong L Batterman S Milando CW. VOC sources and exposures in nail salons: a pilot study in Michigan, USA. Int Arch Occup Environ Health. (2019) 92:141–53. doi: 10.1007/s00420-018-1353-0, PMID: 30276513 PMC6325001

[13] Dang JV Rosemberg MS Le AB. Perceived work exposures and expressed intervention needs among Michigan nail salon workers. Int Arch Occup Environ Health. (2021) 94:2001–13. doi: 10.1007/s00420-021-01719-6, PMID: 34052870 PMC8164489

[14] US Food and Drug Administration. (2013). Nail care products. Available from: http://www.fda.gov/Cosmetics/ProductsIngredients/Products/ucm127068.htm (Accessed 18, May 2015).

[15] Methacrylate Producers Association Inc. (2022). The methacrylate producers association's position on use of methacrylic acid and unreacted methacrylate monomers liquid form in artificial nail products2012 22. Available from: http://static1.1.sqspcdn.com/static/f/1405676/22020353/1361810987690/artificial_nails2.pdf?token=RVAp9IhjiKTwYatLr3EKnTSzkMU%3D (Accessed 22, Nov 2022).

[16] Galloway E Burr G. The nail salon next door. Newsletter. NALBOH Newsbrief. (2006).

[17] Alaves VM Sleeth DK Thiese MS Larson RR. Characterization of indoor air contaminants in a randomly selected set of commercial nail salons in salt Lake County, Utah, USA. Int J Environ Health Res. (2013) 23:419–33. doi: 10.1080/09603123.2012.755152, PMID: 23286453

[18] Quach T Gunier R Tran A von Behren J Doan-Billings PA Nguyen KD. Characterizing workplace exposures in Vietnamese women working in California nail salons. Am J Public Health. (2011) 101:S271–6. doi: 10.2105/AJPH.2010.300099 21551383 PMC3222474

[19] Lamplugh A Harries M Xiang F Trinh J Hecobian A Montoya LD. Occupational exposure to volatile organic compounds and health risks in Colorado nail salons. Environ Pollut. (2019) 249:518–26. doi: 10.1016/j.envpol.2019.03.086, PMID: 30933751

[20] Occupational Safety and Health Administration. Health hazards in nail salons Washington, DC. Available from: https://www.osha.gov/nail-salons/chemical-hazards (Accessed 30, Nov 2022)

[21] Young AS Allen JG Kim U-J Seller S Webster TF Kannan K. Phthalate and organophosphate plasticizers in nail polish: evaluation of labels and ingredients. Environ Sci Technol. (2018) 52:12841–50. doi: 10.1021/acs.est.8b04495, PMID: 30302996 PMC6222550

[22] US Food and Drug Administration. (2013). FDA Authority over cosmetics: How cosmetics are not FDA-approved, but are FDA-regulated. Available from: https://www.fda.gov/cosmetics/cosmetics-laws-regulations/fda-authority-over-cosmetics-how-cosmetics-are-not-fda-approved-are-fda-regulated (Accessed 30, Nov 2022).

[23] Ssempebwa JC Ndejjo R Neebye RM Atusingwize E Musinguzi G. Determinants of exposures to hazardous materials among nail cosmeticians in the Kampala City, Uganda. J Environ Public Health. (2019) 2019:1–9. doi: 10.1155/2019/1925863 PMC6466909 31061662

[24] Hadei M Hopke PK Shahsavani A Moradi M Yarahmadi M Emam B. Indoor concentrations of VOCs in beauty salons; association with cosmetic practices and health risk assessment. J Occup Med Toxicol. (2018) 13:30. doi: 10.1186/s12995-018-0213-x, PMID: 30275872 PMC6161385

[25] Lavoue J. (2015). Expostat—statistical tools for the interpretation of industrial hygiene data. Available from: https://expostats.ca/site/en/tools.html (Accessed 5, May 2022).

[26] Hummel AA Braun KO Fehrenbacher MC. Evaporation of a liquid in a flowing airstream. Am Ind Hyg Assoc J. (1996) 57:519–25. doi: 10.1080/15428119691014729 8651073

[27] Lavoue J. (2015). NDExpo—Treatment of non-detects in industrial hygiene samples. Available from: https://expostats.ca/site/en/othertools.html (Accessed 30, Aug 2022).

[28] Helsel DR. Nondetects and Data Analysis. Statistics for Censored Environmental Data. New York: Wiley (2005).

[29] Goldin LJ Ansher L Berlin A Cheng J Kanopkin D Khazan A. Indoor air quality survey of nail salons in Boston. J Immigr Minor Health. (2014) 16:508–14. doi: 10.1007/s10903-013-9856-y, PMID: 23765035 PMC4008780

[30] Persily A. Development and application of an indoor carbon dioxide metric. Indoor Air. (2022) 32:e13059. doi: 10.1111/ina.13059, PMID: 35904382

[31] Wallenius K Hovi H Remes J Mahiout S Liukkonen T. Volatile organic compounds in Finnish office environments in 2010-2019 and their relevance to adverse health effects. Int J Environ Res Public Health. (2022) 19. doi: 10.3390/ijerph19074411, PMID: 35410093 PMC8999080

[32] Gjølstad M Thorud S Molander P. Occupational exposure to airborne solvents during nail sculpturing. J Environ Monit. (2006) 8:537–42. doi: 10.1039/B601917J, PMID: 16688355

