# Supplementary material for: Exposure of formal and informal nail technicians to organic solvents found in nail products
Source: Front Public Health. 2023 May 4;11:1147204. doi: 10.3389/fpubh.2023.1147204 (PMC10193029; doi:10.3389/fpubh.2023.1147204)
Supplement: Supplementary file 1 [file Table_1.DOCX]

Supplementary Material

**Table S1.** Detected VOCs and their corresponding evaporation rate and weighting ratio.

| **Compound** | **Evaporation rate (mg/min)*** | **Weighting Ratio relative to**  **d-Limonene** | **Evaporation rate weighting factor** |
| --- | --- | --- | --- |
| Ethanol | 37600 | 17.49 | 0.06 |
| Acetone | 136000 | 63.26 | 0.02 |
| Ethyl acetate | 73400 | 35.16 | 0.03 |
| Benzene | 64300 | 32.05 | 0.03 |
| Methyl methacrylate | 428000 | 15.16 | 0.07 |
| Ethyl methacrylate | 18500 | 11.77 | 0.08 |
| Propyl acetate | 28300 | 13.02 | 0.08 |
| Toluene | 23000 | 12.14 | 0.08 |
| n-Butyl acetate | 11200 | 5.21 | 0.19 |
| Xylene | 13100 | 4.12 | 0.24 |
| d-Limonene | 2150 | 1.00 | 1.00 |
| 2-Propanol | 24900 | 11.58 | 0.09 |
| White Spirits | 68800 | 3.20 | 0.31 |

* Evaporation rate calculated by Hummel’s equation

**Table S2.** Within and between informal nail salons variation in Acetone concentrations (n = 10)

| *Variation* | *Sum of squares* | *Degrees of freedom* | *Mean squares* | *F* | *P-value* | *Critical value* |
| --- | --- | --- | --- | --- | --- | --- |
| Between Salons | 11,43240588 | 9 | 1,27026732 | 8,881365153 | 3,77919E-05 | 2,422698937 |
| Within Salons | 2,71749654 | 19 | 0,143026134 |  |  |  |
|  |  |  |  |  |  |  |
| Total | 14,14990242 | 28 |  |  |  |  |

**TABLE S3.** Within and between formal nail salons variation in Acetone concentrations (n = 6)

| *Variation* | *Sum of squares* | *Degrees of freedom* | *Mean squares* | *F* | *P-value* | *Critical value* |
| --- | --- | --- | --- | --- | --- | --- |
| Between Salons | 0,885139456 | 5 | 0,177027891 | 4,083618887 | 0,008447585 | 2,639999426 |
| Within Salons | 0,997066967 | 23 | 0,043350738 |  |  |  |
|  |  |  |  |  |  |  |
| Total | 1,882206423 | 28 |  |  |  |  |

**Table S4:** Pearson’s correlation coefficients and p-values between VOC concentrations, number of clients seen per day and ΔCO_2_ among informal nail salons

|  | Ethanol | Acetone | EA | EMA | Toluene | 2-Propanol | White Spirits | No. of clients | Delta CO_2_ |
| --- | --- | --- | --- | --- | --- | --- | --- | --- | --- |
| Ethanol | 1.0000 |  |  |  |  |  |  |  |  |
| Acetone | 0.2913 | 1.0000 |  |  |  |  |  |  |  |
|  | 0.1252 |  |  |  |  |  |  |  |  |
| EA | -0.0735 | -0.1193 | 1.0000 |  |  |  |  |  |  |
|  | 0.7046 | 0.5375 |  |  |  |  |  |  |  |
| EMA | 0.7732* | 0.3603 | -0.0935 | 1.0000 |  |  |  |  |  |
|  | 0.0000 | 0.0549 | 0.6295 |  |  |  |  |  |  |
| Toluene | 0.3315 | -0.0137 | -0.0888 | 0.0387 | 1.0000 |  |  |  |  |
|  | 0.0790 | 0.9437 | 0.6467 | 0.8422 |  |  |  |  |  |
| 2-Propanol | 0.8007* | 0.1919 | -0.0642 | 0.5492* | 0.2448 | 1.0000 |  |  |  |
|  | 0.0000 | 0.3187 | 0.7408 | 0.0020 | 0.2005 |  |  |  |  |
| White Spirits | 0.2202 | 0.1889 | -0.0210 | 0.2362 | 0.2897 | 0.0363 | 1.0000 |  |  |
|  | 0.2510 | 0.3264 | 0.9138 | 0.2174 | 0.1274 | 0.8519 |  |  |  |
| No. of clients | 0.3748* | 0.5616* | 0.0168 | 0.6634* | -0.0649 | 0.2385 | 0.2585 | 1.0000 |  |
|  | 0.0452 | 0.0015 | 0.9309 | 0.0001 | 0.7380 | 0.2129 | 0.1757 |  |  |
| Delta CO_2_ | 0.2190 | -0.1714 | 0.0759 | -0.0918 | 0.2470 | 0.1036 | -0.0098 | -0.1641 | 1.0000 |
|  | 0.5432 | 0.6359 | 0.8350 | 0.8008 | 0.4914 | 0.7759 | 0.9785 | 0.6506 |  |

*Correlation is significant at the 0.05 level

EA = Ethyl acetate

EMA = Ethyl methacrylate

**TABLE S5:** Pearson’s correlation coefficients and p-value between VOC concentrations, number of clients seen per day and ΔCO_2_ among formal nail salons

|  | Ethanol | Acetone | EA | EMA | Toluene | 2-Propanol | White Spirits | No. of clients | Delta CO_2_ |
| --- | --- | --- | --- | --- | --- | --- | --- | --- | --- |
| Ethanol | 1.0000 |  |  |  |  |  |  |  |  |
| Acetone | 0.0380 | 1.0000 |  |  |  |  |  |  |  |
|  | 0.8450 |  |  |  |  |  |  |  |  |
| EA | 0.5469* | 0.3451 | 1.0000 |  |  |  |  |  |  |
|  | 0.0021 | 0.0667 |  |  |  |  |  |  |  |
| EMA | 0.3504 | -0.1250 | 0.35525 | 1.0000 |  |  |  |  |  |
|  | 0.0624 | 0.5184 | 0.0607 |  |  |  |  |  |  |
| Toluene | -0.0091 | 0.8653* | 0.2269 | -0.0129 | 1.0000 |  |  |  |  |
|  | 0.9627 | 0.0000 | 0.2366 | 0.9471 |  |  |  |  |  |
| 2-Propanol | 0.5261*  0.0034 | 0.2209  0.2495 | 0.5957*  0.0007 | 0.2383  0.2131 | 0.0365  0.8507 | 1.0000 |  |  |  |
| White Spirits | -0.0718  0.7114 | 0.0166  0.9318 | 0.0195  0.9201 | 0.3138  0.0973 | -0.0850  0.6609 | 0.3019  0.1115 | 1.0000 |  |  |
| No. of clients | 0.0239 | -0.1812 | -0.0215 | -0.0874 | -0.2340 | 0.1728 | -0.1565 | 1.0000 |  |
|  | 0.9019 | 0.3470 | 0.9120 | 0.6520 | 0.2219 | 0.3700 | 0.4176 |  |  |
| Delta CO_2_ | -0.1243 | 0.1306 | -0.3644 | -0.2462 | 0.0193 | -0.3086 | -0.0487 | -0.2906 | 1.0000 |
|  | 0.5205 | 0.4993 | 0.0520 | 0.1980 | 0.9210 | 0.1033 | 0.8020 | 0.1262 |  |

*Correlation is significant at the 0.05 level

EA = Ethyl acetate

EMA = Ethyl methacrylate
